# The Targeted Motor Control Screening Tool Is Valid for 4-Year-Old Children

**DOI:** 10.1093/ptj/pzae071

**Published:** 2024-05-14

**Authors:** Laura Brown, Amanda Bacon, Verity Pacey, Emre Ilhan

**Affiliations:** Department of Health Sciences, Faculty of Medicine, Health and Human Sciences, Macquarie University, Sydney, Australia; Movement Mechanics Physiotherapy, Brisbane, Australia; Department of Health Sciences, Faculty of Medicine, Health and Human Sciences, Macquarie University, Sydney, Australia; Department of Health Sciences, Faculty of Medicine, Health and Human Sciences, Macquarie University, Sydney, Australia

**Keywords:** Child, Developmental Disabilities, Early Diagnosis, Pediatrics

## Abstract

**Objective:**

The objective was to determine the validity of the Targeted Motor Control (TMC) screening tool with the Neurosensory Motor Developmental Assessment (NSMDA) in 4-year-old children.

**Methods:**

In this single cohort observational study, children (3 years 9 months to 4 years 5 months) completed the TMC and the NSMDA in a randomized order 5 to 14 days apart.

**Results:**

Seventy-six children (mean age = 4 years 2 months; standard deviation = 2.5 months; *n* = 35 male) completed both assessments. Forty-two children performed within the normal range on the NSMDA. There were significant and positive moderate correlations between the item totals overall and for each area on the NSMDA and the TMC (*r* = 0.40–0.61), and between the NSMDA functional grade for each area and the corresponding TMC areas (*r* = 0.47–0.67). However, the correlation between the NSMDA sensorimotor functional grade and the TMC sensory score was significant but low and positive (*r* = 0.35). The optimal cut-off score for detecting children at risk of atypical development on the TMC was a score of <9 (*n* = 42) (sensitivity = 82.4%; specificity = 66.7%), with a positive likelihood ratio of 2.47 (95% confidence interval [CI] = 1.57–3.89) and a negative likelihood ratio of 0.26 (95% CI = 0.12–0.56).

**Conclusion:**

The TMC is a valid screening tool to identify 4-year-old children at risk of motor delay.

**Impact:**

Early identification of developmental concerns using a validated screening tool is recommended. The TMC is a valid performance-based screening tool that can be used to identify children at risk of atypical motor development who would benefit from further developmental assessment so that, if indicated, timely intervention can be implemented.

## Introduction

Developmental delay occurs when a child is not achieving developmental milestones within the expected timeframe.^1^ Delays can be temporary or ongoing and can occur in social, emotional, motor, language, and/or cognitive function. If developmental delays persist, a child may be diagnosed with a developmental disability or disorder. According to the International Classification of Disability, Functioning and Health, a child with a physical and/or mental impairment has associated activity limitations and participation restrictions, which are compounded by environmental and personal factors.^2^

Developmental coordination disorder (DCD) is an example of a developmental disorder where children have minor motor difficulties that impact on their learning and performance in functional activities, which cannot be explained by the child’s age, intellect, or known physical disorder.^3^^,^^4^ The prevalence of DCD is estimated to be 6% in school-aged children.^5^ Comorbidities are common in children with developmental disorders, such as DCD, and include behavioral conditions (attention deficit hyperactivity disorder, autistic spectrum disorders), altered postural muscle activity, learning difficulties, reduced self-esteem, and social difficulties.^6–9^ Children with DCD may also show widespread alterations in brain structure and function, which may explain the high number of associated comorbidities.^10^ Developmental disorders may also result in lower cardio-respiratory fitness and increased risk of obesity.^11^ Although developmental difficulties, such as motor difficulties experienced by children with DCD may be considered mild, its broader social and economic impacts are significant as the difficulties may continue into adulthood.^12^

Early identification of developmental delay and early access to intervention are recommended to minimize their impact.^13^ Signs of developmental disorders are usually present in early childhood.^12^^,^^14^ Formal diagnosis of some conditions, such as DCD, is not recommended before 5 years of age due to natural variability in trajectories of motor development.^12^ DCD is usually diagnosed between 5 and 16 years of age, although it is often underdiagnosed.^3^^,^^4^ Early identification of developmental delay and intervention during a critical period of neuroplasticity may optimize outcomes for a child by ensuring that the child’s developmental trajectory aligns with that of a typically developing child.^15^^,^^16^ This may also mean that the potential lifelong cascading effects of developmental disorders on a child are minimized and their long-term outcomes are improved.

For these reasons, 4 years of age may be an important timepoint to identify developmental concerns. Ideally, developmental delay should be screened using cost and time-effective methods that require minimal equipment and training.^17^ Children identified as being at risk of developmental delay can then be referred to appropriate health professionals and services for further investigations and potential intervention. As well as assessing development, preschool-aged screening is recommended to assess and facilitate school readiness.^18^ Given school readiness includes the individual, the school, the family, and the community, information about a child’s developmental performance can assist in promoting a smooth transition of the child into the education system.^19^

There are questionnaire-based screening tools that have acceptable levels of sensitivity and specificity at detecting developmental delay.^20^^,^^21^ Performance-based screening tools are also available, including the motor-proficiency-test for children aged 4 to 6 years and the McCarron Assessment of Neuromuscular Development, both of which have been validated against the Movement Assessment Battery for Children^21^; however, the motor-proficiency test for children aged 4 to 6 years has not undergone linguistic validation and translation into English, and the McCarron Assessment of Neuromuscular Development focuses solely on gross and fine motor skills, and not the ability to coordinate motor responses to sensory input as a separate skill. Therefore, there is a lack of validated performance-based screening tools that are applicable in the community setting for 4-year-old children that screen both motor and sensory skills.^21^

Consequently, we developed a simple, quick-to-perform screening tool, the Targeted Motor Control (TMC). The TMC is an abbreviated version of the Neurosensory Motor Developmental Assessment (NSMDA). The NSMDA is a criterion-referenced and standardized assessment that is valid and reliable and characterizes specific areas of motor development.^22^ The NSMDA at 4 years of age has been shown to independently predict motor coordination at 11 to 13 years of age with a positive predictive value of 87%.^23^ However, the NSMDA requires training and experience to administer, and assessments can take up to 45 minutes, which can be time-consuming for an assessor and challenging for a 4-year-old. The TMC was designed to identify children at risk of motor delay (gross motor, fine motor, and sensorimotor such as visual or tactile concerns). However, it may indirectly identify children at risk of delay in other areas of development.

Therefore, the primary aim of our study was to determine the concurrent validity of the TMC when compared to a reference standard, the NSMDA, in children at 4 years of age. The secondary aim was to determine the optimal cut-off value for the TMC to detect children at risk of atypical motor development according to the NSMDA. The final aim was to determine the interrater reliability of the TMC.

## Methods

### Study Design

This was a prospective single cohort observational study.

### Recruitment Strategy

Recruitment occurred between December 2019 and April 2022. Information about the study was advertised using pamphlets and information sheets that were distributed via email, social media platforms, childcare centers, and clinics. A wide recruitment strategy ensured the inclusion of children with and without delay. Children were recruited through childcare centers locally and interstate, health care professionals, and local nongovernment organizations.

### Procedures

Once written informed consent from parents or carers was obtained, the children underwent the NSMDA and the TMC, performed between 5 and 14 days apart. A minimum of 5 days between the NSMDA and the TMC was chosen to avoid a potential learning effect of test items. A maximum of 14 days was chosen to minimize improvement on test items due to maturation.

The order that each child underwent the NSMDA and the TMC was block randomized (*n* = 8 per block). Physical therapists with clinical experience in pediatrics performed all assessments. For each child, the assessors for the NSMDA and the TMC were different to ensure blinding to any performance scores of the children.

Physical therapists who performed the NSMDA had undergone formal training in this assessment (2-day course) prior to assessing children in this study. Physical therapists who performed the TMC had attended a half-day training workshop on the TMC. These physical therapists had also observed and scored 2 prerecorded videos of children undergoing the TMC to ensure consistency in using and scoring the TMC, and to collect data on interrater reliability of TMC assessors. Physical therapists were deemed competent in using the TMC for the study if they scored at a comparable level to an expert in the TMC who scored the same children. Comparable levels were considered to be within 2 points of difference of the expert’s score. It was deemed that a physical therapist was ready to use the TMC independently if they attained this comparable level of scoring.

Children underwent testing at Macquarie University, in their own home, or at the childcare center that they attended. Each child was assessed at the same location for both the NSMDA and the TMC.

### Participants

In order to be included in this study, children were: 3 years 9 months to 4 years 5 months; able to understand and follow verbal instructions in English; not yet attending school; and were able to undergo the NSMDA and the TMC at the same location within 5 to 14 days of each other. If undergoing testing at home, the child’s home had to be within 1-hour drive of Sydney CBD or Brisbane CBD.

Children were excluded if they had any impairments that meant that they could not complete any item on the TMC, such as a neurological impairment, visual impairment not corrected by wearing corrective lenses, hearing impairment not corrected by aids, or a musculoskeletal injury (eg, fracture). A similar approach was taken when determining the eligibility of children with a developmental disability, whereby any impairments that meant a child could not complete any item on the TMC was a basis for exclusion. These exclusion criteria ensured that the performance of children included in this study was based as much on motor skill impairment as possible.

### Measurements

Demographic details collected included the child’s date of birth, sex assigned at birth, and birth history. Parents or carers were also asked to provide details of any relevant medical conditions, and any developmental concerns, and/or therapy that their child was receiving.

#### Neuro-Sensory Motor Developmental Assessment

The NSMDA^22^ rates the child against age-appropriate skills and responses across 6 areas: gross motor, fine motor, infant patterns of movement, postural and balance, neurological and motor responses to sensory input. For each area, a functional grade is assigned ranging from 1, indicating the child is within the normal range for their age, to 5, indicating that the child has no independent function in that particular area. The sum of the functional grades of each area provides an NSMDA total score (range 6–30) and overall motor performance classification: normal/typical motor development (scores 6–8), minimal motor dysfunction (scores 9–11), mild motor dysfunction (scores 12–14), moderate motor disability (scores 15–19), severe disability (scores 20–25), or profound disability (score > 25).^22^

#### Targeted Motor Control

The TMC is based on the NSMDA and has been developed to classify children as either typical or at risk of motor delay. It is an 11-item screening tool as opposed to the 37-item NSMDA. Three factors were considered when selecting items to include in the TMC: (1) What is functionally important for a 4-year-old child? (2) What are the gold standardized tests with age-appropriate scores for this age? (3) What tests are easily administered and require minimal equipment? The TMC takes approximately 15 to 20 minutes to administer and includes age-appropriate tests of gross and fine motor performance, balance and postural control, and sensorimotor function. Examples of gross motor items include balancing on 1 leg, hopping and ball catching, whereas fine motor items include drawing tasks to assess grasp and execution. The sensorimotor area includes an eye follow item to assess the ability of the eyes to track objects independent of head and body movement, fine motor perception tasks to assess light-touch localization and position sense awareness, and a head righting item to assess the ability of the eyes and inner ear to detect head and body movements. Unlike the NSMDA, the TMC does not include items assessing neurological function or infant patterns of movement.

All items on the TMC (except item 5 and item 6) are scored as either 0 (below expected performance for age or an immature response) or +1 (age-appropriate response or above expected response for age). Item 5, head righting, and item 6, ocular motor, are scored using a 3-point ordinal scale, ranging from −1 (well below expected performance for age or a very immature response for age) to +1 (age-appropriate response or above expected response for age). Only these 2 items allow a −1 score to be given to reflect the greater variability in performance on these items. This extra weighting on scoring for these items emphasizes the additional impact that immaturities in these abilities have on age-appropriate function in daily activities. Therefore, the sum of the 11 items gives a total TMC score, ranging from −2 to 11. The TMC items can be grouped into gross motor (items 1–7) and fine motor (items 8–11) areas or motor (items 1–4, 7, 10, 11) and sensory (5, 6, 8, 9) areas.

### Ethical Considerations

This study was approved by the Macquarie University Medicine and Health Sciences Subcommittee (reference no. 52020639314726). Parents and carers consented to publication of study findings.

### Sample Size

The sample size required for this study was based on the recommendations of 2 similar studies.^24^^,^^25^ Hence, a minimum of 66 and a maximum of 80 children completing both assessments were targeted for recruitment to provide a validity estimate that was robust approximately 80% to 100% of the time.

### Statistical Analysis

Data analysis was performed using SPSS version 28.0 (IBM Corp, Armonk, NY, USA). Completion of both the NSMDA and the TMC was required for a child’s data to be included in data analysis. Descriptive statistics was performed for demographic data using means (standard deviation, SD) for parametric continuous variables, and median (range) for non-parametric continuous variables. Categorical variables were summarized using counts (%).

Concurrent validity was determined using Pearson correlation (*r*) when both variables were continuous and Spearman rho (ρ) when 1 variable was ordinal. Only the NSMDA item total and NSMDA functional grade scores were reverse scored to align with the direction of the TMC scores. The strength of the correlation coefficient was considered none to very low (0–0.25), low (0.26–0.40), moderate (0.41–0.69), high (0.70–0.89), and very high (0.90–1).^26^

The optimal cut-off value of the TMC was determined using receiver operating characteristic curve analysis, with the NSMDA as the reference standard, with a functional grade of 6–8 corresponding to typical development, and any grade above 8 considered atypical development. An area under the curve of 0.50 indicated no discriminative ability, 0.70 to 0.80 as being acceptable, 0.80 to 0.90 as being excellent, and 0.90 to 1 as being outstanding.^27^ The Youden index (J)^28^ was used to determine the threshold of optimal detection of at risk of atypical versus typical motor development.

Interrater reliability was determined by calculating intraclass correlation coefficients (95% CI) based on a 2-way mixed effects model, with a mean rating (*k* = 6; *n* = 2) and agreement for consistency (intraclass correlation coefficient = 3.1). Interrater reliability was based on the TMC total scores. An intraclass correlation coefficient of between 0.50 and 0.75 demonstrated moderate reliability, 0.75 to 0.90 good reliability, and values more than 0.90 excellent reliability.^29^

### Role of the Funding Source

The funders played no role in the design, conduct, or reporting of this study.

## Results

**Figure 1:**
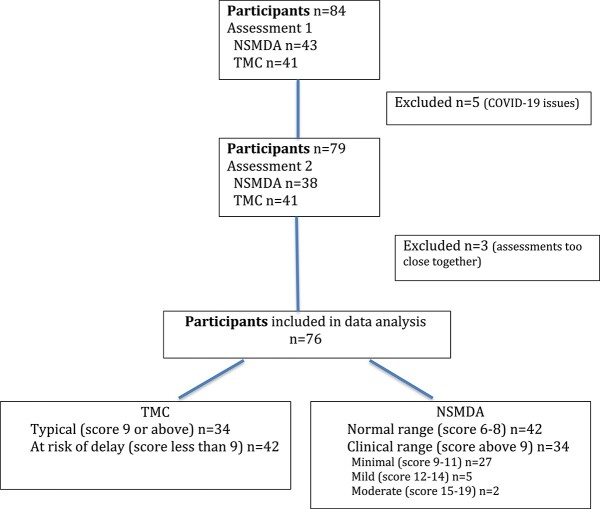
Flow of participants. NSMDA = Neurosensory Motor Developmental Assessment; TMC = Targeted Motor Control

Seventy-six (*n* = 35 male) participants completed both the NSMDA and the TMC. Therefore, 76 paired results were included in the analyses (Fig. 1). Reasons for participants not completing both the NSMDA and the TMC are summarized in Figure 1.

The mean age of participants that completed both the NSMDA and the TMC was 4 years 2 months (SD = 2.5 months) at assessment 1 (Tab. 1). Demographic details of the participants are described in Table 1.

### Concurrent Validity

The mean (SD; range) of the total score for the TMC was 7.2 (SD = 2.6; range = 1–11), and for the NSMDA was 9.0 (SD = 2.1; range = 6–16). Tables 2 and 3 report the correlation coefficients between scores on the TMC and the NSMDA. There were significant positive correlations between the item totals overall and for each area on the NSMDA and the TMC which were moderate (range in *r* = 0.40–0.61); however, the correlation between the NSMDA sensorimotor item total and the TMC sensory score was significant and positive but low (*r* = 0.40). There was a significant and positive moderate correlation between the functional grade of each area of the NSMDA and their corresponding areas on the TMC (range in ρ = 0.47–0.67). The correlation between the sensorimotor functional grade of the NSMDA sensorimotor functional grade and the TMC sensory score was significant and positive but low (ρ = 0.35).

### Optimal Cut-off Value for the TMC to Detect Being at Risk of Atypical Motor Development

Receiver operating characteristic curve analysis revealed an area under the curve of 0.80 (95% CI = 0.70–0.90; *P* < .001). This indicated that the TMC had an acceptable to excellent level of discrimination between those with and without at least an atypical level of development according to the NSMDA.

**Figure 2:**
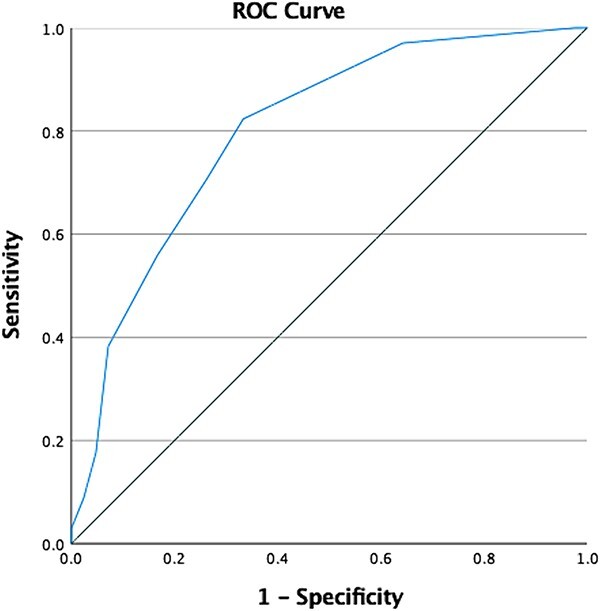
Receiver operating characteristic (ROC) curve. This ROC curve represents the optimal cut-off score (<9) in detecting children at risk of atypical development, which was associated with a sensitivity of 0.824, and a specificity of 0.667. The area under the curve was 0.801

Analysis of the coordinates of the curve and calculation of Youden J demonstrated the optimal cut-off score for detecting at least being at risk of an atypical level of motor development on the TMC was a score of <9 (sensitivity = 82.4%; specificity = 66.7%), which had a positive likelihood ratio of 2.47 (95% CI = 1.57–3.89) and a negative likelihood ratio of 0.26 (95% CI = 0.12–0.56) (Fig. 2). The false positive rate for this cut-off score was 33.3%. Finally, the positive and negative predictive values for the TMC were 75% and 76%, respectively. For comparability with the NSMDA, the total TMC scores below 9 were thus termed “at-risk of atypical motor development” and scores 9 and above were termed typical for motor development. The term at risk highlights that the TMC is a screening tool.

### Interrater Reliability of the TMC

The interrater reliability was assessed across 6 assessors who all observed and scored the 2 prerecorded videos of children undergoing the TMC. Intraclass correlation coefficient analysis (3.1) revealed an intraclass correlation coefficient of 0.87 (95% CI = 0.34–1) using a single measures estimate, which demonstrated a good level of interrater reliability (*F*_1,5_ = 40.69; *P* = .001).

## Discussion

Our study demonstrated a moderate level of concurrent validity of the TMC when compared to the NSMDA in 4-year-old children. The concurrent validity between the sensory area on the TMC and the sensorimotor area on the NSMDA, however, was low. The optimal cut-off value for the TMC to detect children at risk of atypical motor development was a score of less than 9. We showed that there was an acceptable level of interrater reliability for the TMC.

The TMC demonstrated an adequate test accuracy (area under the curve > 0.70) and sensitivity (>80%), but the specificity was below population-based screening recommendations.^21^ Children with signs of developmental delay require continual assessment of their skills over time to account for the natural variability in skill development. Therefore, lower levels of test specificity may be more acceptable compared to the standard set for other screening tests such as cancer^21^ that often require high levels of specificity for tests that are invasive and expensive. Additionally, the concurrent validity between gross motor and fine motor areas on the TMC and the NSMDA in this study is important, especially as the concurrent validity of screening tests in other studies has been weak.^21^ Similarities between gross motor and fine motor areas on the tests may be reflective of the TMC providing an accurate and balanced level of representation of NSMDA items in these areas. Our finding of low concurrent validity between the sensory area on the TMC and the sensorimotor area on the NSMDA may be related to differences in the number and range of items in this area between the tests. Specifically, the NSMDA captures a broader range and greater number of sensorimotor items compared to the TMC sensory area. The imbalance of items in this area between the screening tool and the reference standard has impacted validity outcomes in other studies.^21^

**Table 1:** Demographic Details of Participants*^a^*

**Demographic Details**	**Participants (*n* = 76)**
Age at NSMDA assessment (mean, mo)	4 y 2 mo, SD = 2.5 (*n* = 72*^b^*)
Sex assigned at birth (*n*)	Male *n* = 35; Female *n* = 41
Mean birthweight (grams)	3213 (*n* = 44*^b^*)
Premature (*n*)	7 (*n* = 63*^b^*)
Mean gestational age, if premature	34 wk 5 d
Medical conditions*^c^* (*n*)	11 (*n* = 63*^b^*)
Developmental concerns (*n*)	7 (*n* = 63*^b^*)
Therapy*^d^*	8 (*n* = 63*^b^*)

^a^ NSMDA = Neurosensory Motor Developmental Assessment.

^b^ Demographic detail based on this number of participants (ie, missing data).

^c^ Medical conditions: cardiac (*n* = 1), respiratory (*n* = 1), orthopedic (*n* = 5), musculoskeletal (*n* = 1), neurological (*n* = 1), cleft lip, and palate and/or grommets (*n* = 2).

^d^ Therapy included occupational therapy (*n* = 3), speech therapy (*n* = 6), physical therapy (*n* = 3), podiatry (*n* = 1). Note of the 8 children receiving therapy, 3 children were receiving multiple therapies (2 therapies [*n* = 2], 3 therapies [*n* = 1]).

A cut-off value of <9 on the TMC may be fitting given that the purpose of the TMC as a screening tool is to identify those children who may be at risk of motor delay. Therefore, as appropriate, children who score <9 can be referred to services for comprehensive assessment. Indeed, the TMC was originally developed to be used by pediatric health professionals, highlighting the importance of detecting any motor concern rather than just moderate to severe delay. A more conservative cut-off value minimizes the risk of health professionals underrecognizing mild delay, which commonly occurs due to the subtlety of the signs of mild delay.^30^

Additionally, given the simple and easy-to-use nature of the TMC, there is potential for it to be used by non-health professionals who work with children in the future. This extended reach to non-health professionals provides further justification for using a more conservative cut-off value on the TMC when considering referral for further assessment. Non-health professionals with varying levels of developmental screening experience, such as early childhood educators, may be ideally placed to screen for children with mild developmental delay but are not able to perform complex and lengthy performance-based assessments. Additional work to enable early childhood educators to reliably use the TMC is needed. As part of this process, it may be necessary to consider concerns that early childhood educators may have to support them in using this screening tool.

**Table 2:** Correlation Matrix Between Item Total Scores of the Targeted Motor Control (TMC) and the Neurosensory Motor Developmental Assessment (NSMDA) Using Pearson Correlation

**Pearson Correlation, *r* (95% CI)**	**NSMDA–Item Total Gross Motor**	**NSMDA–Item Total Fine Motor**	**NSMDA–Item Total Sensorimotor**	**NSMDA–Item Total** ^***a***^
TMC—Gross motor	.56*^b^**^,^**^c^* (0.38–0.70)	.33*^c^* (0.11–0.52)	.40*^c^* (0.20–0.58)	.56*^c^* (0.38–0.70)
TMC—Fine motor	.26*^d^* (0.03–0.46)	.61*^b^**^,^**^c^* (0.44–0.73)	.44*^c^* (0.24–0.60)	.42*^c^* (0.22–0.59)
TMC—Sensory	.26*^d^* (0.04–0.46)	.36*^c^* (0.19–0.57)	.40*^b^**^,^**^c^* (0.19–0.57)	.36*^c^* (0.15–0.54)
TMC—Total	0.52*^c^* (0.33–0.67)	0.53*^c^* (0.35–0.68)	0.50*^c^* (0.31–0.65)	.60*^b^**^,^**^c^* (0.43–0.73)

^a^ NSMDA–item total was reversed scored.

^b^ Correlations refer to the concurrent validity.

^c^ Significance (2-tailed) = <0.005.

^d^ Significance (2-tailed) = <0.05.

**Table 3:** Correlation Matrix Between Domains of the Targeted Motor Control (TMC) and the Functional Grades of Areas on the Neurosensory Motor Developmental Assessment (NSMDA) and Areas on the TMC Using Spearman Rho

**Spearman Rho, *r* (95% CI)**	**NSMDA—Functional Gross Motor** ^***a***^	**NSMDA—Functional Fine Motor** ^***a***^	**NSMDA—Functional Sensorimotor** ^***a***^
TMC—Gross motor	.47*^b^**^,^**^c^* (0.30–1.00)	.41*^c^* (0.20–0.60)	.40*^c^* (0.18–0.58)
TMC—Fine motor	.19 (−0.04 to 0.41)	.67*^b^**^,^**^c^* (0.54–1)	.32*^d^* (0.10–0.51)
TMC—Sensory	.11 (−0.13 to 0.33)	.40 (0.18–0.58)*^c^*	.35*^b^**^,^**^c^* (0.17–1)

^a^ NSMDA functional grade was reversed scored.

^b^ Correlations refer to the concurrent validity.

^c^ Significance (2-tailed) = <0.005.

^d^ Significance (2-tailed) = <0.05.

Another reason for being overly cautious with 4-year-old children relates to the role of parents or carers in their child’s development. If a child is at risk of delay, it is important to alert parents or carers immediately prior to their child commencing formal education. Furthermore, the results of the TMC may provide objective information for parents or carers about their child at a critical time at which point early intervention may be most beneficial. Although the TMC targets children transitioning into formal education, in some cases, children will be outside the age range for the TMC (3 years 9 months to 4 years 5 months). In time, TMC screening tools for children older or younger than 4 years of age may be developed. The TMC in its current form has been validated against the NSMDA at 4 years, hence the strict age band for its use.

We have provided evidence of the concurrent validity of the TMC and we have established an optimal cut-off value of <9 as indicating a child at risk of atypical motor development. The identification of children who may have at least a mild developmental delay prior to the commencement of formal education is important to enable timely access to intervention.^13^ Four years of age is a particularly critical age because, if intervention is required, it is an optimal timepoint for it as it aligns with neuroplasticity^15^^,^^16^ and responsiveness to change.

## Data Availability

The data supporting the findings of this study are available from the corresponding author upon request.
